# Supplementary material for: Recurrent Herpes Simplex Virus Type 1 (HSV-1) Infection Modulates Neuronal Aging Marks in In Vitro and In Vivo Models
Source: Int J Mol Sci. 2021 Jun 11;22(12):6279. doi: 10.3390/ijms22126279 (PMC8230621; doi:10.3390/ijms22126279)
Supplement: Supplementary file 1 [file ijms-22-06279-s001.zip › ijms-1206182-supplementary.pdf]

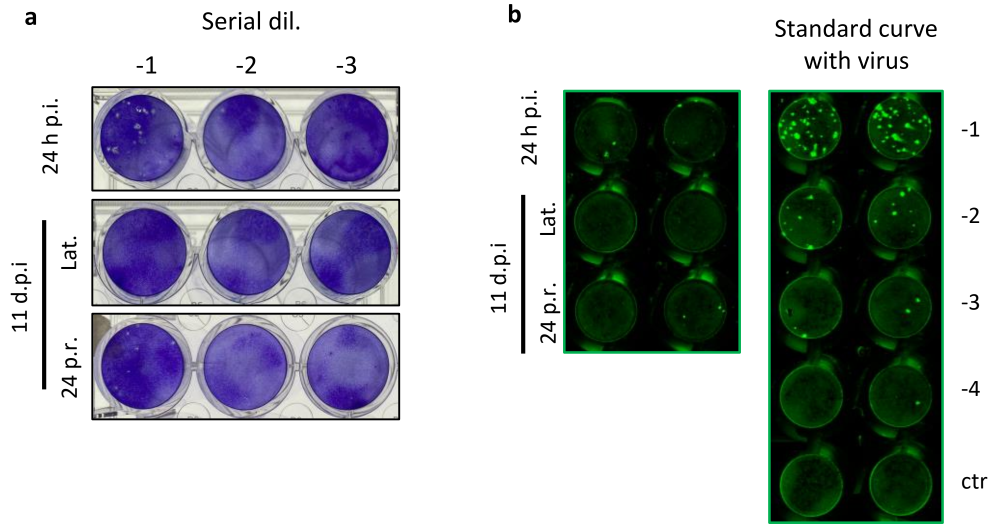

c

Sample	Viral titer (SPA)	Viral titer (ICW) *FFU (gB-detected HSV-1 foci)
24 h p.i	8,5 x 10^2 PFU/ml	8 x 10^1 FFU*/ml
11 d.p.i. Lat	0 PFU/ml	0 FFU/ml
11 d.p.i. 24 h p.r	1,16 x 10^2 PFU/ml	3 x 10^1 FFU/ml

Figure S1. Virus titration in cell supernatants from the in vitro model of recurrent HSV-1 infection. Cell supernatants were harvested from neurons infected with 0.1 M.O.I. of HSV-1 for 24 h (24 h p.i.), 11 days in the presence of ACV (11 d.p.i. LAT) and 24 h post reactivation (11 d.p.i. 24 h p.r.) as described in matherials and methods. **a)** images of Standard Plaque Assay (SPA) of a representative experiment; **b)** representative images of In Cell Western showing gb-stained HSV-1 foci of the same samples titred in panel a (left image) and of a reference standard curve of HSV-1 virus (right panel). **c)** virus titers of samples from the representative experiment showed in panel a and b
